# A mathematical model of cartilage regeneration after chondrocyte and stem cell implantation – I: the effects of growth factors

**DOI:** 10.1177/2041731419827791

**Published:** 2019-03-15

**Authors:** Kelly Campbell, Shailesh Naire, Jan Herman Kuiper

**Affiliations:** 1 School of Computing and Mathematics, Keele University, Keele, UK; 2 Institute for Science and Technology in Medicine, Keele University, Keele, UK; 3 The Robert Jones and Agnes Hunt Orthopaedic Hospital NHS Foundation Trust, Oswestry, UK

**Keywords:** Mathematical modelling, cartilage defect, regenerative medicine

## Abstract

Autologous chondrocyte implantation is a cell-based therapy for treating chondral defects. The procedure begins by inserting chondrocytes into the defect region. The chondrocytes initiate healing by proliferating and depositing extracellular matrix, which allows them to migrate into the defect until it is completely filled with new cartilage. Mesenchymal stem cells can be used instead of chondrocytes with similar long-term results. The main differences are at early times since mesenchymal stem cells must first differentiate into chondrocytes before cartilage is formed. To better understand this repair process, we present a mathematical model of cartilage regeneration after cell therapy. We extend our previous work to include the cell–cell interaction between mesenchymal stem cells and chondrocytes via growth factors. Our results show that matrix formation is enhanced at early times in the presence of growth factors. This study reinforces the importance of mesenchymal stem cell and chondrocyte interaction in the cartilage healing process as hypothesised in experimental studies.

## Introduction

Developing and improving upon the treatment of articular cartilage damage is a fundamental clinical problem. Articular cartilage damage occurs in several ways, from playing high-contact sport to natural wear and tear, affecting a variety of different age groups and sexes. The ability of damaged cartilage to self-repair is limited due to its avascularity and can often lead to osteoarthritis when left untreated.^1,2^ Almost nine million people in the United Kingdom are affected by osteoarthritis, which carries a lifetime risk in the knee of approximately 45%.^3,4^

**Figure 1.**
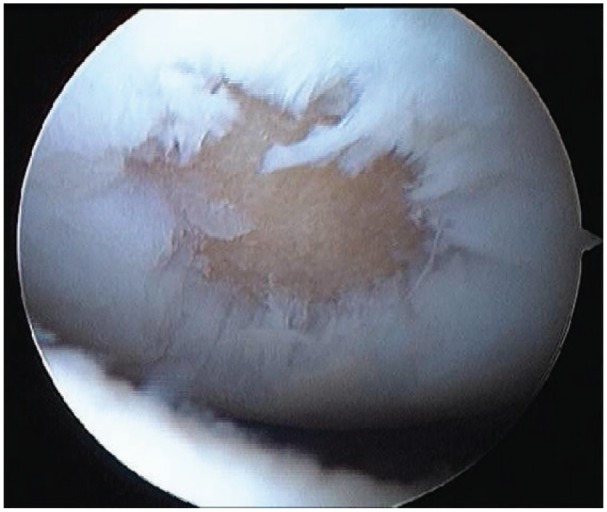
Arthroscopic image of a cartilage defect in the knee.

**Figure 2.**
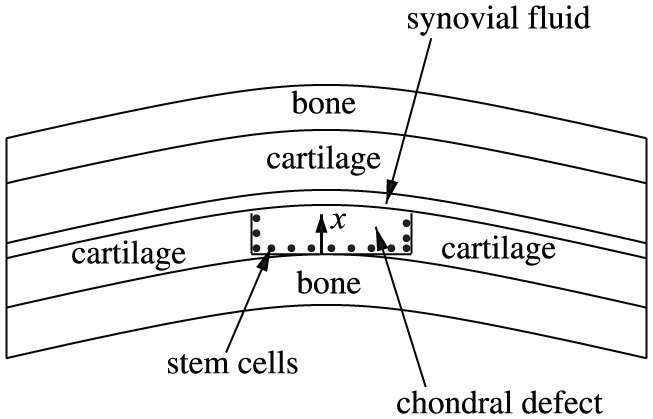
Schematic representation of a cross section of the defect. After debridement of the defect, either chondrocytes or MSCs are seeded along the defect walls.

Autologous chondrocyte implantation (ACI) is a commonly used cell-based therapy mainly used in the treatment of cartilage damage in the knee, first implemented clinically in 1987.^5^ The treatment involves obtaining chondrocytes from a biopsy of healthy cartilage, culturing and expanding these chondrocytes in vitro for several weeks to an amount in excess of 5–10 million,^6^ and a surgical implantation procedure of these cultured cells into the damaged (or defect) region.^5,7^ An alternate cell-based therapy, which we refer to as articular stem cell implantation (ASI), replicates the ACI procedure except that mesenchymal stem cells (MSCs) are used instead of chondrocytes.^8^ The capacity of stem cells to differentiate into different cell types along with their abundance within the body and the ease with which they can be harvested makes them advantageous to be used in cell-based therapies instead of chondrocytes. Figures 1 and 2 show a cartilage defect in the knee and a schematic of the defect cross section, respectively. The diameter of the defect is about 10–20 mm and its thickness is about 2–3 mm. After debridement of the defect, chondrocytes or MSCs are implanted into the defect along the bottom and sides. The initial number of cells implanted are around 10^6^ cells/cm^2^ of the defect area.^9^

The chondrocytes proliferate (by taking up nutrients) and migrate, in the process forming extracellular matrix (ECM) and new cartilage. In the case of MSCs, the process of forming new cartilage is initiated only after the stem cells first differentiate into chondrocytes. ECM comprises primarily water, proteoglycans such as GAGs (glycosaminoglycans) and proteins such as collagens. Chondrocytes sit within lacunae in the deepest layers of ECM and as such have limited motility within the matrix, giving rise to the poor reparative ability of articular cartilage.^10^ ECM also acts as a structural component of cartilage and provides important mechanical properties.^11^ The mechanical stresses generated by loading or unloading the knee joint, for example, can influence ECM production and hence the tissue’s overall structure by modulating the cell proliferation, differentiation and migration rates.^12^ Growth factors, such as those from the transforming growth factor-beta (TGF-β) family, for example, TGF-β1 and bone morphogenetic protein (BMP-2), and fibroblast growth factor, FGF-1 and FGF-2, are also known to regulate cell migration, proliferation and differentiation, although their mechanisms are not clearly understood.

**Figure 3.**
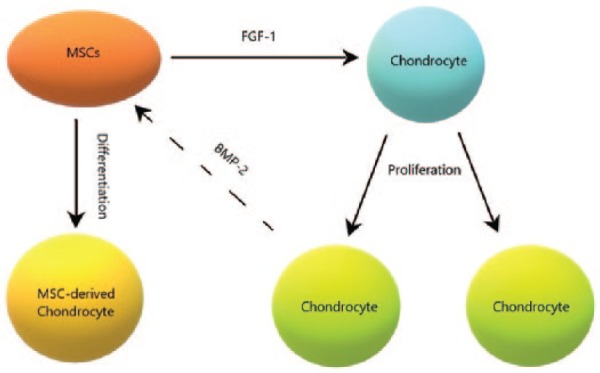
Schematic representation of hypothesised cross-talking between chondrocytes and MSCs mediated by FGF-1 and BMP-2. Source: Adapted from Wu.^13^

Recently, Wu^13^ investigated the role of growth factors in a co-culture of stem cells and chondrocytes in vitro. Their findings show that when culturing a mixture of stem cells and chondrocytes, an increase in matrix deposition is observed. This increase can be approximately quantified to be 50% for a 50:50 ratio of MSCs to chondrocytes, and 30% for an 80:20 ratio in comparison with a 100% MSC seeding at 4 weeks. This increase in matrix deposition was attributed to specific growth factors produced by the stem cells and chondrocytes. They identified the growth factors to be BMP-2 and FGF-1. FGF-1 is produced by the MSCs and is shown to influence the proliferation of the chondrocyte population. On the other hand, BMP-2 is produced by the chondrocytes and is shown to induce chondrogenesis of MSCs. These two growth factors are hypothesised to mediate the mutual chondrocyte and MSC interaction as shown in Figure 3. This hypothesis assumes that the increased matrix production is explained by the increased number of chondrocytes due to the actions of both growth factors. The same authors also found evidence that FGF-1 leads to increased matrix production per chondrocyte, which could also explain the increased matrix deposition in their experiments.

In humans, many details of tissue regeneration after surgical cell implantation are unknown. The only detailed data currently available are from magnetic resonance imaging (MRI) scans and 1-year biopsies which show the condition of the cartilage.^14^ Some insight into cartilage healing can be obtained from animal models.^15^ Characterising the success of the surgery is closely linked to the structural composition of the regenerated tissue.^16^ However, there is little information on the cell-to-cell interactions which lead to the development and regeneration of the tissue. In this respect, theoretical models of tissue regeneration have contributed significantly to the understanding of the cell-to-cell interactions and other contributing factors influencing the healing process.

In our previous work,^17^ a mathematical model was formulated to describe the various processes involved in the regeneration of a cartilage defect following the implantation of chondrocytes (ACI) or MSCs (ASI). Our model showed that during the healing process, there is very little difference in the overall time taken to heal the defect between the two cell therapies, suggesting that regeneration using stem cells alone is no better or worse than that using chondrocytes. The stem cells need to first differentiate into chondrocytes before forming ECM and new cartilage, a process that is initiated only after the stem cell density exceeds a threshold value. The overall healing time frame of about 18 months for the defect to reach full maturation corresponds with results from clinical studies and demonstrated that cartilage regeneration is a slow process. The only stem cell–chondrocyte interaction considered in this work was the one-way interaction in which MSCs differentiated to form chondrocytes once a threshold stem cell density was exceeded. This work did not include the influence of growth factors, as well as MSC–chondrocyte interaction. Related modelling studies have highlighted the importance of growth factors and MSC–chondrocyte interactions. Kimpton et al.^18^ showed how different cell seeding strategies and growth factors effect the spatial distribution of cells within a hydrogel inserted into a chondral defect. Chen et al.^19^ explored the interactions between MSCs, chondrocytes and TGF-β. They demonstrated how adopting this strategy combining growth factors produced by the cells and exogeneous addition of growth factors has advantages over each individual strategy. More relevant to our work is the mathematical model of fracture healing by Bailon-Plaza and van der Meulen.^20^ They demonstrated the mediating effects of BMP-2 and TGF-β1 on the chondrocyte–osteoblast interaction and their influence on the bone regeneration during fracture healing.

In the literature, there are an abundance of theoretical models for tissue regeneration, taking either a discrete or continuum approach to modelling. We formulate our model using a continuum approach due to the high cell densities used in the surgical procedure (above a few million). Sherratt and Murray^21^ present a reaction–diffusion mathematical model on epidermal wound healing, using their results to validate that biochemical regulation is a key mechanism in wound regeneration. Olsen et al.^22^ assess the ECM involvement in tumour angiogenesis using a standard continuum modelling framework. Other examples of continuum reaction–diffusion-type equations can be found in the works by Bailon-Plaza and van der Meulen^20^ and Obradovic et al.^23^ Using these modelling approaches as motivation, we formulate our model using reaction–diffusion-type equations. This allows us to model migration of cells as a diffusive process and the differentiation, proliferation and death of cells as reaction terms. This modelling approach also allows for the uptake of nutrients by the cells to initiate proliferation, with nutrient uptake rates modelled following Michaelis–Menten kinetics. As in our previous work,^17^ we focus our modelling on ECM production via stem cell differentiation to chondrocytes and chondrocyte proliferation. Cell behaviour is regulated by nutrients available within the defect, which in our case is oxygen. Cell proliferation and differentiation are influenced by the growth factors. We consider FGF-1 and BMP-2, which we subsequently anticipate will effect matrix deposition.

The primary focus of this article, encouraged by the findings of Wu,^13^ is to investigate the role of growth factors and MSC–chondrocyte interactions in the regeneration of cartilage after stem cell implantation (ASI). Once stem cells differentiate into chondrocytes, we can expect to see the same cell-to-cell interaction observed in co-cultures of MSCs and chondrocytes with similar trophic effects.^13^ In the second part, we consider a co-implantation of MSCs and chondrocytes to see how this impacts matrix deposition compared with ACI and ASI cell therapies, motivated by a potentially earlier healing time. To achieve this, we first seek to address the specific question of the impact of growth factors, released via cell-to-cell interaction, on the deposition of matrix during chondral healing. Co-implantation of MSCs and chondrocytes could have important implications on how clinicians approach surgical procedures of the regeneration of cartilage, indicating that a potentially superior procedure could be implemented involving a mixture of MSCs and chondrocytes. We extend our previous model^17^ to include the actions of growth factors BMP-2 and FGF-1 and investigate their mediating role on chondrocyte and MSC interaction hypothesised by Wu^13^ and shown in Figure 3. Including their proposed stem cell–chondrocyte interaction into our model would also enable validation of the enhanced matrix levels observed.^13^

The plan of the article is as follows. In the ‘Mathematical model’ section, we describe the basic model and the assumptions made, the boundary and initial conditions used, estimates of the parameter values and the scalings used to non-dimensionalise the equations. The results of our simulations are discussed in the ‘Results’ section, for two cases in which no, either or both growth factors are present and their comparison. Results showing sensitivity to certain parameters are also shown here. Finally, in the ‘Discussion’ section, we examine the implications of the model results on ACI therapy and future work.

## Mathematical model

### Formulation

A typical catilage defect has a small thickness depth to length ratio. This enables us to simplify to a one-dimensional problem where cell growth is modelled along the defect thickness only, shown as the *x*-direction in Figure 2. The variables in our model are as follows: the stem cell density, $C_{S}$ (cells/mm^3^), chondrocyte density, $C_{C}$ (cells/mm^3^), matrix density, *m* (g/mm^3^), nutrient concentration, *n* (moles/mm^3^), FGF-1 concentration, *g* (g/mm^3^) and BMP-2 concentration, *b* (g/mm^3^).

We follow the model of Lutianov et al.^17^ to describe the evolution of the cell and matrix densities and nutrient concentration in time, *t*, and space, *x*, measured along the thickness of the defect (see Figure 2). We state with brief comments the equations and refer the interested reader to Lutianov et al.^17^ for further details of modelling choices. We focus on the evolution of the growth factor concentrations FGF-1 and BMP-2 and their coupling to the chondrocyte proliferation and stem cell differentiation, respectively.

The rate of change of stem cell density, based on proliferation by uptake of nutrients, migration and differentiation into chondrocytes, is modelled as follows

(1) $$\begin{array}{c} \frac{\partial C_{S}}{\partial t}=\frac{\partial }{\partial x}\left(D_{S}(m)\frac{\partial C_{S}}{\partial x}\right)\\ +p_{1}\left(m,\frac{C_{S}}{C_{S,\max}(m)}\right)\frac{n}{n+n_{0}}C_{S}H(n-n_{1})\\ -p_{2}C_{S}H(C_{S}-C_{S_{0}}(b))-p_{3}C_{S}H(n_{1}-n) \end{array}$$

The third term on the right of equation (1) models stem cell differentiation into chondrocytes at a rate $p_{2}$ (assumed constant). This process is initiated once $C_{S}$ exceeds a threshold density $C_{S_{0}}$ modelled using the Heaviside function $H(C_{S}-C_{S_{0}})$, which takes the unit value when $C_{S}>C_{S_{0}}$ and zero otherwise. We assume that the BMP-2 growth factor concentration modulates stem cell differentiation by reducing the threshold density and is modelled as follows

(2) $$C_{S_{0}}(b)=(C_{S_{0,\max}}-C_{S_{0,\min}})e^{-\alpha b}+C_{S_{0,\min}}$$

where $C_{S_{0,\max}}$ and $C_{S_{0,\min}}$ are maximum and minimum threshold densities, respectively, and *α* is a decay constant. Alternatively, one could also model this modulation by making the stem cell differentiation rate, $p_{2}$, dependent on the BMP-2 growth factor concentration, keeping $C_{S_{0}}$ fixed. We do not consider this here but briefly mention any sensitivity to this in the ‘Sensitivity of parameters and initial conditions’ section. The first, second and fourth terms on the right of equation (1) model stem cell migration (modelled as a diffusion process), proliferation and cell death, respectively, where $D_{s}$ is the stem cell random motility (diffusion) coefficient (assumed to depend on the matrix density), $p_{1}$ is the stem cell proliferation rate (assumed to depend on the matrix and stem cell densities) and $p_{3}$ is the stem cell death rate (assumed constant). Following Lutianov et al.,^17^ we choose

$$\begin{array}{l} D_{S}(m)=D_{S_{0}}\frac{m}{m^{2}+m_{1}^{2}}\\ p_{1}\left(m,\frac{C_{S}}{C_{S,\max}(m)}\right)=A(m)\left(1-\frac{C_{S}}{C_{S,\max}(m)}\right)\\ A(m)=p_{1_{0}}\frac{m}{m^{2}+m_{2}^{2}}\\ C_{S,\max}(m)=C_{S,\max_{0}}\left(1-\frac{m}{m_{\max}}\right) \end{array}$$

where $D_{S_{0}}$ and $p_{1_{0}}$ are reference migration and proliferation rates, respectively; $m_{1}$ and $m_{2}$ are reference matrix densities; and $C_{S,\max_{0}}$ and $m_{\max}$ are the maximum stem cell and matrix density, respectively. Diffusion is modelled to be dependent on the matrix density, as done in the related literature.^20^ Cell motility is expected to increase for lower matrix densities and decrease for higher densities (see the work by Lutianov et al.^17^ for full details).

Similar to the above, the rate of change of chondrocyte density is modelled as follows

(3) $$\begin{array}{c} \frac{\partial C_{C}}{\partial t}=\frac{\partial }{\partial x}\left(D_{C}(m)\frac{\partial C_{C}}{\partial x}\right)\\ +p_{4}\left(m,g,\frac{C_{C}}{C_{C,\max}(m)}\right)\frac{n}{n+n_{0}}C_{C}H(n-n_{1})\\ +p_{2}C_{S}H(C_{S}-C_{S_{0}}(b))-p_{5}C_{C}H(n_{1}-n) \end{array}$$

where $D_{C}$ is the chondrocyte random motility (diffusion) coefficient, $p_{4}$ is the chondrocyte proliferation rate and $p_{5}$ is the chondrocyte death rate. We use similar expressions as above for

$$\begin{array}{l} D_{C}(m)=D_{C_{0}}\frac{m}{m^{2}+m_{1}^{2}}\\ p_{4}\left(m,g,\frac{C_{C}}{C_{C,\max}(m)}\right)=B(m,g)\left(1-\frac{C_{C}}{C_{C,\max}(m)}\right)\\ B(m,g)=\left(p_{4_{0}}\frac{m}{m^{2}+m_{2}^{2}}+p_{4_{00}}\frac{g}{g+g_{0}}\right)\\ C_{C,\max}(m)=C_{C,\max0}\left(1-\frac{m}{m_{\max}}\right) \end{array}$$

where $D_{C_{0}}$ is the reference diffusion rate, $p_{4_{0}}$ is the reference proliferation rate, $m_{1}$ and $m_{2}$ are reference matrix densities and $C_{C,\max_{0}}$ is the maximum chondrocyte density (see the work by Lutianov et al.^17^ for details). The additional contribution to chondrocyte proliferation due to the influence of the FGF-1 growth factor is modelled by the second term in the expression for B(m,g) in equation (4). Here, $p_{4_{00}}$ and $g_{0}$ are the reference proliferation rate and FGF-1 concentration, respectively (Table 1). When *g* is small, $p_{4_{00}}(g/g+g_{0})$ increases linearly, saturating to a limiting value of $p_{4_{00}}$ for larger values of *g*. A similar term representing the effect of growth factors on proliferation is used by Bailon-Plaza and van der Meulen^20^ and replicates a Michaelis–Menten-type saturation term. We assume the biological effect of the growth factor is an additive contribution to that from the matrix density; hence, we add it to the original proliferation term, $p_{4_{0}}(m/m^{2}+m_{2}^{2})$.

**Table 1.** Estimated values of dimensional parameters.

Dimensional parameters	Estimated value
*d*, defect thickness	2 mm
$D_{S}$, maximum stem cell migration (or diffusion) coefficient	3.6 × (10^−4^–10^−3^) mm^2^/h ^23^
$D_{C}$, maximum chondrocyte migration (or diffusion) constant	3.6 × 10^−4^ mm^2^/h ^23^
$D_{S_{0}}=2m_{1}D_{S}$, stem cell migration (or diffusion) constant,	7.2 × (10^−9^–10^−8^) (mm^2^/h) (g/mm^3^) (assuming $m_{1}=10^{-5}\;{g/\mathrm{mm}}^{3}$)
$D_{C_{0}}=2m_{1}D_{C}$, chondrocyte migration (or diffusion) constant,	7.2 × 10^−9^ (mm^2^/h) (g/mm^3^) (assuming $m_{1}=10^{-5}\;{g/\mathrm{mm}}^{3}$)
$D_{n}$, nutrient diffusion coefficient	4.6 mm^2^/h^24^
$D_{m}$, matrix diffusion coefficient	2.5 × 10^−5^ mm^2^/h ^23^
$D_{g}$, FGF-1 diffusion coefficient	2 × 10^−3^ mm^2^/h ^20^
$D_{b}$, BMP-2 diffusion coefficient	2 × 10^−3^ mm^2^/h ^20^
$p_{1}$, maximum stem cell proliferation rate	0.2 cell/h or 5 cells/day^20^
$p_{1_{0}}=2m_{2}p_{1}$^17^ stem cell proliferation constant	$4\times 10^{-6}\;{g/\mathrm{mm}}^{3}/h$ (assuming $m_{2}=10^{-5}\;{g/\mathrm{mm}}^{3}$)
$p_{2}$, stem cell differentiation rate	3.75 × 10^−3^/h ^23^
$p_{3}s$, stem cell death rate	3.75 × 10^−3^/h (guess)
$p_{4}$, maximum chondrocyte proliferation rate	2 × 10^−4^/h (guess)
$p_{4_{0}}=2m_{2}p_{4}$,^17^ chondrocyte proliferation constant	4 × 10^−9^ g/mm^3^/h
$p_{5}$, chondrocyte death rate	3.75 × 10^−3^/h (guess)
$p_{9}$, FGF-1 production constant	10^−17^ (g/mm^3^)/((Nc/mm^3^) h) (guess)
$p_{11}$, FGF-1 degradation rate	5.8 × 10^−2^/h (based on 12 h half-life guess)
$p_{12}$, BMP-2 production constant	10^−17^ (g/mm^3^)/((Nc/mm^3^) h) (guess)
$p_{13}$, BMP-2 degradation rate	5.8 × 10^−2^/h (based on 12 h half-life)
$p_{4_{00}}$, chondrocyte proliferation rate (from FGF-1)	2 × 10^−4^/h (guess)
$p_{8_{0}}$, matrix production constant	3.75 × 10^−13^ (g/mm^3^)/((Nc/mm^3^) h)^23^
$p_{8_{1}}$, matrix degradation constant	3.75 × 10^−13^ (g/mm^3^)/((Nc/mm^3^) h)^23^
$p_{6}$, nutrient uptake constant by stem cells	1.5 × 10^−14^ N m/(Nc h)^24^
$p_{7}$, nutrient uptake constant by chondrocytes	1.5 × 10^−14^ Nm/(Nc h)^24^
$p_{8_{00}}$, FGF-1 matrix deposition rate	0–1 (guess)
$C_{total,\max_{0}}$, maximum total cell density	$10^{6}\;{\mathrm{Nc}/\mathrm{mm}}^{3}$ (assuming 10mm10μm cell diameter)
$C_{S,\max_{0}}$, maximum stem cell density	$0-10^{6}\;{\mathrm{Nc}/\mathrm{mm}}^{3}$
$C_{C,\max_{0}}$, maximum chondrocyte density	$0-10^{6}\;{\mathrm{Nc}/\mathrm{mm}}^{3}$
$m_{\max}$, maximum matrix density	$10^{-4}\;{g/\mathrm{mm}}^{3}$ ^20^
$C_{S}^{\left(0\right)}$, initial stem cell density	$2.5\times 10^{5}\;{\mathrm{Nc}/\mathrm{mm}}^{3}$ (based on $10^{6}$ cells in 20 mm × 20 mm × 10 µm volume)
$C_{C}^{\left(0\right)}$, initial cartilage cell density	$10^{2}\;{\mathrm{Nc}/\mathrm{mm}}^{3}$ ($10^{-2}\%$ of total cell density)
$C_{S_{0\max}}$, threshold stem cell density	$C_{total,\max_{0}}/2$ Nc/mm^3^ (guess)
$C_{S_{0\min}}$, threshold stem cell density	90% of $C_{S_{0\max}}$ (guess)
$m_{1}$, matrix density	$10^{-5}\;{g/\mathrm{mm}}^{3}$ (assumed $m_{\max}/10$)^17^
$m_{2}$, matrix density	$10^{-5}\;{g/\mathrm{mm}}^{3}$ (assumed $m_{\max}/10$)^17^
$m_{3}$, initial matrix density	$10^{-8}\;{g/\mathrm{mm}}^{3}$ (assumed $m_{\max}/10^{4}$)^17^
$N_{0}$, initial nutrient concentration	$(2.85-9.5)\times 10^{-11}\;{\mathrm{Nm}/\mathrm{mm}}^{3}$ ^24^
$g_{init}$, initial FGF-1 concentration	10^−12^ g/mm^3 20^
$b_{init}$, initial BMP-2 concentration	10^−12^ g/mm^3 20^
$n_{0}$, threshold nutrient concentration	$2.3\times 10^{-11}\;{\mathrm{Nm}/\mathrm{mm}}^{3}$ ^24^
$n_{1}$, critical nutrient concentration	$9.5\times 10^{-12}\;{\mathrm{Nm}/\mathrm{mm}}^{3}$ (assumed $N_{0}/10$)
*α*, threshold stem cell density reduction factor	10^10^/(g/mm^3^) (guess)
$g_{0}$, FGF-1 reference concentration	10^−10^ g/mm^3 20^
$b_{0}$, BMP-2 reference concentration	10^−10^ g/mm^3 20^
γ, FGF-1 flux coefficient	$10^{-2}$ mm/h (guess)
χ, BMP-2 flux coefficient	$10^{-2}$ mm/h (guess)

In the above, $N_{C}$ represents number of cells and $N_{m}$ is number of moles.

The rate of change of nutrient concentration and matrix density are as given with full modelling justification in the work by Lutianov et al.^17^ with minor changes made to our *m* equation. The rate of change of nutrient concentration is modelled by a Fickian-type diffusion term with nutrient uptake terms proportional to chondrocyte and stem cell densities, with a Michaelis–Menten-type nutrient saturation. The rate of change of matrix density similarly comprises a diffusion term, a production term proportional to the chondrocyte density that is limited by a Michaelis–Menten-type nutrient saturation term

(4) $$\frac{\partial n}{\partial t}=D_{n}\frac{\partial^{2}n}{\partial x^{2}}-\frac{n}{n+n_{0}}(p_{6}C_{S}+p_{7}C_{C})$$

(5) $$\frac{\partial m}{\partial t}=D_{m}\frac{\partial^{2}m}{\partial x^{2}}+p_{8}(m,g)\frac{n}{n+n_{0}}C_{C}$$

where $D_{n}$ and $D_{m}$ are the nutrient and matrix diffusion coefficients, respectively (assumed constant); $n_{0}$ is the reference nutrient concentration, $p_{6}$ and $p_{7}$ represent the nutrient uptake rate by stem cells and chondrocytes, respectively (assumed constant); and $p_{8}(m,g)=(p_{8_{0}}-p_{8_{1}}m)(1+p_{8_{00}}(g/g+g_{0}))$ is the matrix synthesis rate, where $p_{8_{0}}$ is the matrix production rate, $p_{8_{1}}$ is the matrix degradation rate and the last term in the brackets accounts for any additional matrix directly produced by FGF-1 with a pre-factor $0<p_{8_{00}}<1$. The main effect of FGF-1 is thought to be indirectly through the increase in chondrocyte proliferation modelled in equation (3). For our simulations, we set $p_{8_{00}}=0$ and explore the effects of non-zero values of $p_{8_{00}}$ in the ‘Sensitivity of parameters and initial conditions’ section.

The growth factor FGF-1 is produced by the stem cells; it migrates along the defect, degrades and then diffuses out of the upper end of the defect. Using this information, we model the rate of change of FGF-1 as follows

(6) $$\frac{\partial g}{\partial t}=D_{g}\frac{\partial^{2}g}{\partial x^{2}}+p_{9}C_{S}-p_{11}g$$

The first term on the right of equation (6) models diffusion of FGF-1 along the defect, with $D_{g}$ (assumed constant) representing its diffusion coefficient. The second term on the right of equation (6) models the production of FGF-1, assumed proportional to the stem cell density, with production rate $p_{9}$. The third term on the right of equation (6) models the degradation of FGF-1 at a constant rate $p_{11}$.

The growth factor BMP-2 is produced by the chondrocytes; it can migrate along the defect and degrades. Using this information, we model the rate of change of BMP-2 as follows

(7) $$\frac{\partial b}{\partial t}=D_{b}\frac{\partial^{2}b}{\partial x^{2}}+p_{12}C_{C}-p_{13}b$$

The first term on the right of equation (7) models diffusion of BMP-2 along the defect, with $D_{b}$ (assumed constant) representing its diffusion coefficient. The second term on the right of equation (7) models the production of BMP-2, assumed proportional to the chondrocyte density, with production rate $p_{12}$. The third term on the right of equation (7) models the degradation of BMP-2 at a constant rate $p_{13}$.

### Boundary conditions

We need to prescribe two boundary conditions for each variable. These boundary conditions are specified at the lower end of the defect, *x* = 0 (subchondral bone interface), and upper end of the defect, *x* = *d* (normal cartilage surface), where *d* is the thickness of the defect. At x=0, we impose no flux of cells, matrix, nutrients and growth factors, that is

(8) $$\begin{array}{l} -D_{S}(m)\frac{\partial C_{s}}{\partial x}=-D_{C}(m)\frac{\partial C_{c}}{\partial x}=-D_{n}\frac{\partial n}{\partial x}\\ =-D_{m}\frac{\partial m}{\partial x}=-D_{g}\frac{\partial g}{\partial x}=-D_{b}\frac{\partial b}{\partial x}=0 \end{array}$$

At x=d we impose

(9) $$\begin{array}{l} -D_{S}(m)\frac{\partial C_{s}}{\partial x}=-D_{C}(m)\frac{\partial C_{c}}{\partial x}=-D_{m}\frac{\partial m}{\partial x}=0\\ n=N_{0},\;-Dg\frac{\partial g}{\partial x}=\gamma g,\;-Db\frac{\partial b}{\partial x}=\chi b \end{array}$$

The first, second and third boundary conditions represent no flux of stem cells, chondrocytes and matrix, respectively, from the normal cartilage interface. We assume that a reservoir of nutrients with concentration, $N_{0}$, is always available at this end. A small flux of growth factors FGF-1 and BMP-2 are allowed to diffuse out of the defect and is modelled to be proportional to the respective growth factor concentrations with constants of proportionality, *γ* and *χ* (assumed constant). A sensitivity analysis has been performed on *γ* and *χ* in the ‘Sensitivity of parameters and initial conditions’ section, with their approximate values given in Table 1.

### Initial conditions

The initial conditions at t=0 are prescribed as follows

(10) $$\begin{array}{l} C_{S}=C_{S}^{\left(0\right)}h(x),\;C_{C}=C_{C}^{\left(0\right)},\;n=N_{0},\;m=m_{3}\\ g=g_{init},\;b=b_{init} \end{array}$$

Here, $C_{S}^{\left(0\right)}$ and h(x) are an initial stem cell density and profile, respectively. $C_{C}^{\left(0\right)}$, $m_{3}$, $g_{init}$ and $b_{init}$ are some initial chondrocyte and matrix densities and growth factor concentrations (assumed to be uniformly distributed in the defect). The initial nutrient concentration is uniform with value $N_{0}$. The values of $C_{S}^{\left(0\right)},C_{C}^{\left(0\right)},N_{0},m_{3},g_{init},andb_{init}$ are stated and referenced in Table 1.

### Non-dimensionalisation

There are several dimensional parameters appearing in the model. Their estimated values and the references from which they are obtained are provided in Table 1. All approximated parameters are disclosed in the table and references are given where available.

We non-dimensionalise equations (1)–(10) by introducing the following dimensionless variables based on characteristic quantities for each variable

(11) $$\begin{array}{l} \bar{x}=x/d,\;\bar{t}=t(p_{8_{0}}C_{total,\max_{0}}/m_{\max})\\ ({\bar{C}}_{S},{\bar{C}}_{C})=(C_{S},C_{C})/C_{total,\max_{0}},\;\bar{m}=m/m_{\max}\\ \bar{n}=n/N_{0},\;\bar{g}=g/g_{0},\;\bar{b}=b/b_{0} \end{array}$$

where the overbars represent dimensionless quantities. The characteristic quantities used to measure the spatial variable, *x*; cell densities, matrix density and nutrient and growth factor concentrations are the defect thickness, *d*; the reference maximum total cell density, $C_{total,\max0}$; the maximum matrix density, $m_{\max}$; the initial nutrient concentration, $N_{0}$; and reference growth factor concentrations, $g_{0}$ and $b_{0}$, respectively. We choose to measure time, *t*, based on the matrix production time scale, $m_{\max}/(p_{8_{0}}C_{total,\max_{0}})$. Using the parameter values in Table 1, we estimate this time scale to be approximately 11 days. Henceforth, a unit of time corresponds to approximately 11 days. For each variable, a sensitivity analysis was undertaken by increasing and decreasing their tabulated values given in Table 2 and investigating the effect on matrix production.

**Table 2.** Estimated values of dimensionless parameters.

Dimensionless parameters	Estimated value
Stem cell migration (or diffusion) constant, ${\bar{D}}_{S_{0}}=D_{S_{0}}/(p_{8_{0}}C_{total,\max_{0}}d^{2})$	10^−3^–10^−2^
Chondrocyte migration (or diffusion) constant, ${\bar{D}}_{C_{0}}=D_{C_{0}}/(p_{8_{0}}C_{total,\max_{0}}d^{2})$	10^−3^
Nutrient diffusion coefficient, ${\bar{D}}_{n}=D_{n}m_{\max}/(p_{8_{0}}C_{total,\max_{0}}d^{2})$	$(1-3)\times 10^{2}$
Matrix diffusion coefficient, ${\bar{D}}_{m}=D_{m}/(p_{8_{0}}C_{total,\max_{0}}d^{2})$	10^−3^–10^−2^
FGF-1 diffusion coefficient, ${\bar{D}}_{g}=D_{g}m_{\max}/(p_{8_{0}}C_{total,\max_{0}}d^{2})$	1.14
BMP-2 diffusion coefficient, ${\bar{D}}_{b}=D_{b}m_{\max}/(p_{8_{0}}C_{total,\max_{0}}d^{2})$	1.14
Stem cell proliferation constant, ${\bar{p}}_{1_{0}}=p_{1_{0}}/(p_{8_{0}}C_{total,\max_{0}})$	12
Stem cell differentiation rate, ${\bar{p}}_{2}=p_{2}m_{\max}/(p_{8_{0}}C_{total,\max_{0}})$	1
Stem cell death rate, ${\bar{p}}_{3}=p_{3}m_{\max}/(p_{8_{0}}C_{total,\max_{0}})$	1
Chondrocyte proliferation constant, ${\bar{p}}_{4_{0}}=p_{4_{0}}/(p_{8_{0}}C_{total,\max_{0}})$	0.012
Chondrocyte death rate, ${\bar{p}}_{5}=p_{5}m_{\max}/(p_{8_{0}}C_{total,\max_{0}})$	1
FGF-1 production constant, ${\bar{p}}_{9}=p_{9}m_{\max}/(p_{8_{0}}g_{0})$	26.67
FGF-1 degradation rate, ${\bar{p}}_{11}=p_{11}m_{\max}/(p_{8_{0}}C_{total,\max_{0}})$	15.4
BMP-2 production constant, ${\bar{p}}_{12}=p_{12}m_{\max}/(p_{8_{0}}b_{0})$	26.67
BMP-2 degradation rate, ${\bar{p}}_{13}=p_{13}m_{\max}/(p_{8_{0}}C_{total,\max_{0}})$	15.4
Chondrocyte proliferation rate (from FGF-1), ${\bar{p}}_{4_{00}}=p_{4_{00}}m_{\max}/(p_{8_{0}}C_{total,\max_{0}})$	0.012
Matrix degradation constant, ${\bar{p}}_{8_{1}}=p_{8_{1}}m_{\max}/p_{8_{0}}$	1
Nutrient uptake constant by stem cells, ${\bar{p}}_{6}=p_{6}m_{\max}/(p_{8_{0}}N_{0})$	10^4^
Nutrient uptake constant by chondrocytes, ${\bar{p}}_{7}=p_{7}m_{\max}/(p_{8_{0}}N_{0})$	10^4^
FGF-1 matrix deposition rate, ${\bar{p}}_{8_{00}}$	0–1
Threshold nutrient concentration, ${\bar{n}}_{0}=n_{0}/N_{0}$	0.24–0.81
Critical nutrient concentration, ${\bar{n}}_{1}=n_{1}/N_{0}$	0.1
Threshold stem cell density, ${\bar{C}}_{S_{0\max}}=C_{S_{0\max}}/C_{total,\max_{0}}$	0.35
Threshold stem cell density, ${\bar{C}}_{S_{0\min}}=C_{S_{0\min}}/C_{total,\max_{0}}$	0.315
Initial stem cell density, ${\bar{C}}_{S}^{\left(0\right)}=C_{S}^{\left(0\right)}/C_{total,\max_{0}}$	0.25
Initial chondrocyte density, ${\bar{C}}_{C}^{\left(0\right)}=C_{C}^{\left(0\right)}/C_{total,\max_{0}}$	10^4^
Maximum stem cell density, ${\bar{C}}_{S,\max_{0}}=C_{S,\max_{0}}/C_{total,\max_{0}}$	0.6
Maximum chondrocyte density, ${\bar{C}}_{C,\max_{0}}=C_{C,\max_{0}}/C_{total,\max_{0}}$	0.4
Matrix density, ${\bar{m}}_{1}=m_{1}/m_{\max}$	$10^{-1}$
Matrix density, ${\bar{m}}_{2}=m_{2}/m_{\max}$	$10^{-1}$
Initial matrix density, ${\bar{m}}_{3}=m_{3}/m_{\max}$	$10^{-4}$
Initial FGF-1 concentration, ${\bar{g}}_{init}=g_{init}/g_{0}$	$10^{-2}$
Initial BMP-2 concentration, ${\bar{b}}_{init}=b_{init}/b_{0}$	$10^{-2}$
FGF-1 flux coefficient, $\bar{\gamma }=\gamma /(p_{8_{0}}C_{total,\max_{0}}d/m_{\max})$	1
BMP-2 flux coefficient, $\bar{\chi }=\chi /(p_{8_{0}}C_{total,\max_{0}}d/m_{\max})$	1
Threshold stem cell density reduction factor, $\bar{\alpha }=\alpha b_{0}$	100

The dimensionless equations using the above non-dimensionalisation are as follows

(12a) $$\begin{array}{l} \frac{\partial {\bar{C}}_{S}}{\partial \bar{t}}=\frac{\partial }{\partial \bar{x}}\left({\bar{D}}_{S}(\bar{m})\frac{\partial {\bar{C}}_{S}}{\partial \bar{x}}\right)\\ +{\bar{p}}_{1}\left(\bar{m},\frac{{\bar{C}}_{S}}{{\bar{C}}_{S,\max}(\bar{m})}\right)\frac{\bar{n}}{\bar{n}+{\bar{n}}_{0}}{\bar{C}}_{S}H(\bar{n}-{\bar{n}}_{1})\\ -{\bar{p}}_{2}{\bar{C}}_{S}H({\bar{C}}_{S}-{\bar{C}}_{S_{0}}(\bar{b}))\\ -{\bar{p}}_{3}{\bar{C}}_{S}H({\bar{n}}_{1}-\bar{n}) \end{array}$$

(12b) $$\begin{array}{l} \frac{\partial {\bar{C}}_{C}}{\partial \bar{t}}=\frac{\partial }{\partial \bar{x}}\left({\bar{D}}_{C}(\bar{m})\frac{\partial {\bar{C}}_{C}}{\partial \bar{x}}\right)\\ +{\bar{p}}_{4}\left(\bar{m},\bar{g},\frac{{\bar{C}}_{C}}{{\bar{C}}_{C,\max}(\bar{m})}\right)\frac{\bar{n}}{\bar{n}+{\bar{n}}_{0}}{\bar{C}}_{C}H(\bar{n}-{\bar{n}}_{1})\\ +{\bar{p}}_{2}{\bar{C}}_{S}H({\bar{C}}_{S}-{\bar{C}}_{S_{0}}(\bar{b}))\\ -{\bar{p}}_{5}{\bar{C}}_{C}H({\bar{n}}_{1}-\bar{n}) \end{array}$$

(12c) $$\frac{\partial \bar{n}}{\partial \bar{t}}={\bar{D}}_{n}\frac{\partial^{2}\bar{n}}{\partial {\bar{x}}^{2}}-\frac{\bar{n}}{\bar{n}+{\bar{n}}_{0}}({\bar{p}}_{6}{\bar{C}}_{S}+{\bar{p}}_{7}{\bar{C}}_{C})$$

(12d) $$\frac{\partial \bar{m}}{\partial \bar{t}}={\bar{D}}_{m}\frac{\partial^{2}\bar{m}}{\partial {\bar{x}}^{2}}+{\bar{p}}_{8}(\bar{m},\bar{g})\frac{\bar{n}}{\bar{n}+{\bar{n}}_{0}}{\bar{C}}_{C}$$

(12e) $$\frac{\partial \bar{g}}{\partial \bar{t}}={\bar{D}}_{g}\frac{\partial^{2}\bar{g}}{\partial {\bar{x}}^{2}}+{\bar{p}}_{9}{\bar{C}}_{S}-{\bar{p}}_{11}\bar{g}$$

(12f) $$\frac{\partial \bar{b}}{\partial \bar{t}}={\bar{D}}_{b}\frac{\partial^{2}\bar{b}}{\partial {\bar{x}}^{2}}+{\bar{p}}_{12}{\bar{C}}_{C}-{\bar{p}}_{13}\bar{b}$$

where

(13) $$\begin{array}{l} {\bar{p}}_{1}\left(\bar{m},\frac{{\bar{C}}_{S}}{{\bar{C}}_{S,\max}(\bar{m})}\right)=\bar{A}(\bar{m})\left(1-\frac{{\bar{C}}_{S}}{{\bar{C}}_{S,\max}(\bar{m})}\right)\\ \bar{A}(\bar{m})={\bar{p}}_{1_{0}}\frac{\bar{m}}{{\bar{m}}^{2}+{\bar{m}}_{2}^{2}}\\ {\bar{p}}_{4}\left(\bar{m},\bar{g},\frac{{\bar{C}}_{C}}{{\bar{C}}_{C,\max}(\bar{m})}\right)=\bar{B}(\bar{m},\bar{g})\left(1-\frac{{\bar{C}}_{C}}{{\bar{C}}_{C,\max}(\bar{m})}\right)\\ \bar{B}(\bar{m},\bar{g})={\bar{p}}_{4_{0}}\frac{\bar{m}}{{\bar{m}}^{2}+{\bar{m}}_{2}^{2}}+{\bar{p}}_{4_{00}}\frac{\bar{g}}{\bar{g}+1}\\ {\bar{C}}_{S,\max}(\bar{m})={\bar{C}}_{S,\max_{0}}(1-\bar{m})\\ {\bar{C}}_{C,\max}(\bar{m})={\bar{C}}_{C,\max_{0}}(1-\bar{m})\\ {\bar{C}}_{S,\max_{0}}+{\bar{C}}_{C,\max_{0}}=1\\ {\bar{p}}_{8}(\bar{m},\bar{g})=(1-{\bar{p}}_{8_{1}}\bar{m})\left(1+{\bar{p}}_{8_{00}}\frac{\bar{g}}{\bar{g}+1}\right)\\ {\bar{D}}_{S}(\bar{m})={\bar{D}}_{S_{0}}\frac{\bar{m}}{{\bar{m}}^{2}+{\bar{m}}_{1}^{2}},\;{\bar{D}}_{C}(\bar{m})={\bar{D}}_{C_{0}}\frac{\bar{m}}{{\bar{m}}^{2}+{\bar{m}}_{1}^{2}}\\ {\bar{C}}_{S_{0}}(\bar{b})=({\bar{C}}_{S_{0,\max}}-{\bar{C}}_{S_{0,\min}})e^{-\bar{\alpha }\bar{b}}+{\bar{C}}_{S_{0,\min}} \end{array}$$

The non-dimensional boundary and initial conditions are as follows

(14a) $$\begin{array}{l} -{\bar{D}}_{S}(\bar{m})\frac{\partial {\bar{C}}_{S}}{\partial \bar{x}}=-{\bar{D}}_{C}(\bar{m})\frac{\partial {\bar{C}}_{C}}{\partial \bar{x}}=-{\bar{D}}_{n}\frac{\partial \bar{n}}{\partial \bar{x}}\\ =-{\bar{D}}_{m}\frac{\partial \bar{m}}{\partial \bar{x}}=-{\bar{D}}_{g}\frac{\partial \bar{g}}{\partial \bar{x}}=-{\bar{D}}_{b}\frac{\partial \bar{b}}{\partial \bar{x}}=0\;(\mathrm{at}\;\bar{x}=0) \end{array}$$

(14b) $$\begin{array}{l} -{\bar{D}}_{S}(\bar{m})\frac{\partial {\bar{C}}_{S}}{\partial \bar{x}}=-{\bar{D}}_{C}(\bar{m})\frac{\partial {\bar{C}}_{C}}{\partial \bar{x}}=-{\bar{D}}_{m}\frac{\partial \bar{m}}{\partial \bar{x}}=0\\ \bar{n}=1,\;-{\bar{D}}_{g}\frac{\partial \bar{g}}{\partial \bar{x}}=\bar{\gamma }\bar{g},\;-{\bar{D}}_{b}\frac{\partial \bar{b}}{\partial \bar{x}}=\bar{\chi }\bar{b}\;(\mathrm{at}\;\bar{x}=1) \end{array}$$

(14c) $$\begin{array}{l} {\bar{C}}_{S}={\bar{C}}_{S}^{\left(0\right)}\bar{h}(\bar{x}),\;{\bar{C}}_{C}={\bar{C}}_{C}^{\left(0\right)}\bar{h}(\bar{x}),\;\bar{n}=1,\;\bar{m}={\bar{m}}_{3}\\ \bar{g}={\bar{g}}_{init},\;\bar{b}={\bar{b}}_{init},\;(\mathrm{at}\;\bar{t}=0) \end{array}$$

The dimensionless parameters and their estimated values are provided in Table 2.

## Results

We use a second-order accurate finite difference discretisation scheme to discretise the spatial variable *x* in equations (12)–(14), keeping the time derivative *t* continuous. The resulting ordinary differential equations are solved in MATLAB (Release 2013a; The MathWorks, Inc., Natick, MA, USA) using the stiff ODE solver *ode15s*. We refer the reader to Table 2 for information on parameter values.

We first consider the case where the defect is only seeded with stem cells and there are no growth factors present. These results will be used as a baseline case to compare with the case which includes the influence of growth factors. We re-run these simulations from Lutianov et al.,^17^ where a flux of MSCs entering from the bottom of the defect, thought to be sourced by the surrounding defect, was considered. Here, we omit this flux, as clinical guidelines state the underlying subchondral bone of a chondral defect is to be left intact, meaning we would not necessarily observe this flux.^7^

**Figure 4.**
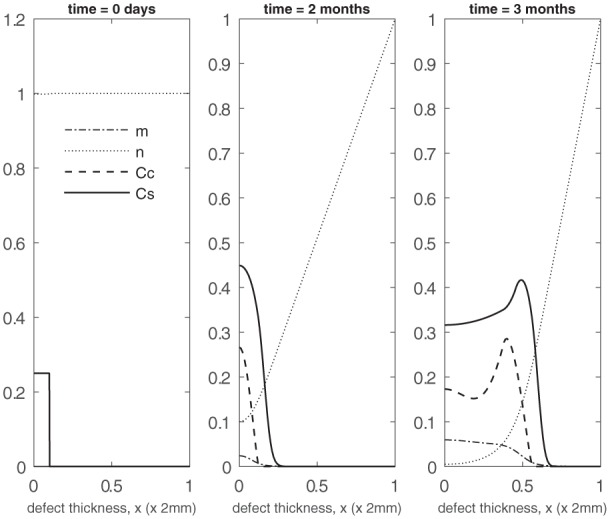
Evolution of cell and matrix densities and nutrient concentration at *t* = 0 days, 2 months, 3 months.

**Figure 5.**
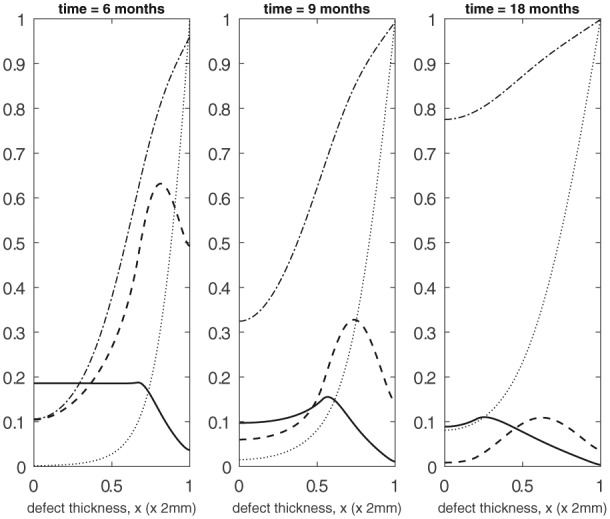
Evolution of cell and matrix densities and nutrient concentration at *t* = 6, 9 and 18 months.

Initially, stem cells are seeded close to the subchondral bone side of the defect (x=0), and the nutrient concentration is uniform (Panel 1 in Figure 4). The nutrient we consider in our model is oxygen, assumed to be diffusing in from the surrounding synovium of the joint. We also assume a small density of chondrocytes and matrix $\left({\bar{C}}_{C}^{\left(0\right)}={\bar{m}}_{3}=10^{-4}\right)$ uniformly distributed across the defect in order to activate the cell and matrix evolution. Figures 4 and 5 show the evolution of the stem cell density, $C_{S}$ (×10^6^ cells/mm^3^), chondrocyte density, $C_{C}$ (×10^6^ cells/mm^3^), matrix density, *m* (×10^4^ g/mm^3^) and nutrient concentration, *n* (×10^−11^ moles/mm^3^), for time ranging between 2 and 18 months. Over the first few days (not shown here), the initial seeding of stem cells start to proliferate by taking up nutrients resulting in a gradual decline of nutrients near x=0. The stem cells are also observed to slowly diffuse away from this end. Up until 2 months, the stem cells have not yet proliferated enough to exceed their differentiation threshold value $\left({\bar{C}}_{S_{0}}=0.35\right)$. As a result, there are no chondrocytes formed from stem cell differentiation and hence there is no matrix deposition. From approximately 2 months onwards, the stem cells have now exceeded their threshold value near x=0 and we observe rapid formation of chondrocytes which in turn increases the matrix deposition at a rapid rate (Panel 2 in Figure 4). We observe the formation of two fronts in the stem cell and chondrocyte densities which gradually migrate up the defect where a higher concentration of nutrients is available (Panel 3 in Figure 4). The stem cell density front migrates faster than the chondrocyte front owing to its higher diffusion coefficient.^23^ We also observe that when the nutrient concentration surpasses its critical value, there is a peak in stem cells, and as a result a peak in chondrocyte density (Panels 2 and 3 in Figure 4). The peak in chondrocytes is also due to the stem cells exceeding their differentiation threshold. Over the course of the first few months, we clearly observe an increase in matrix levels (Panels 2 and 3 in Figure 4). At later times (4 months and beyond), an increase in matrix density is observed near the upper end of the defect due to this increase in chondrocyte formation observed from the peak in stem cells. This is enabled by the large amount of nutrients available (Panel 1 in Figure 5). From approximately 9 months onwards, matrix production continues gradually filling up the entire defect from the upper end down (Panels 2 and 3 in Figure 5).

**Figure 6.**
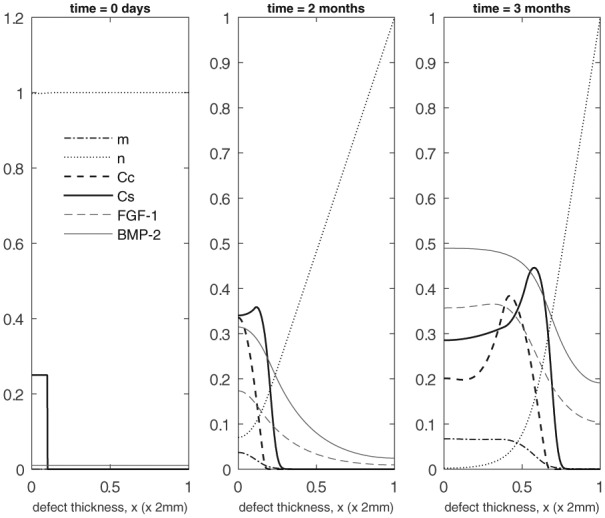
Evolution of cell and matrix densities and nutrient and growth factor concentrations at *t* = 0 days, 2 months, 3 months.

**Figure 7.**
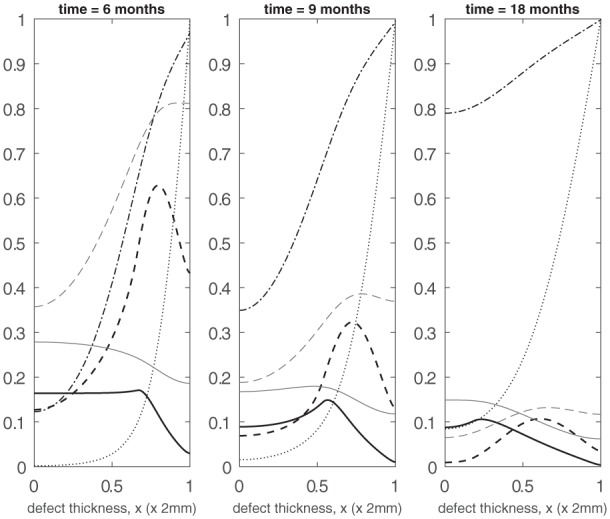
Evolution of cell and matrix densities and nutrient and growth factor concentrations at *t* = 6, 9 and 18 months.

**Figure 8.**
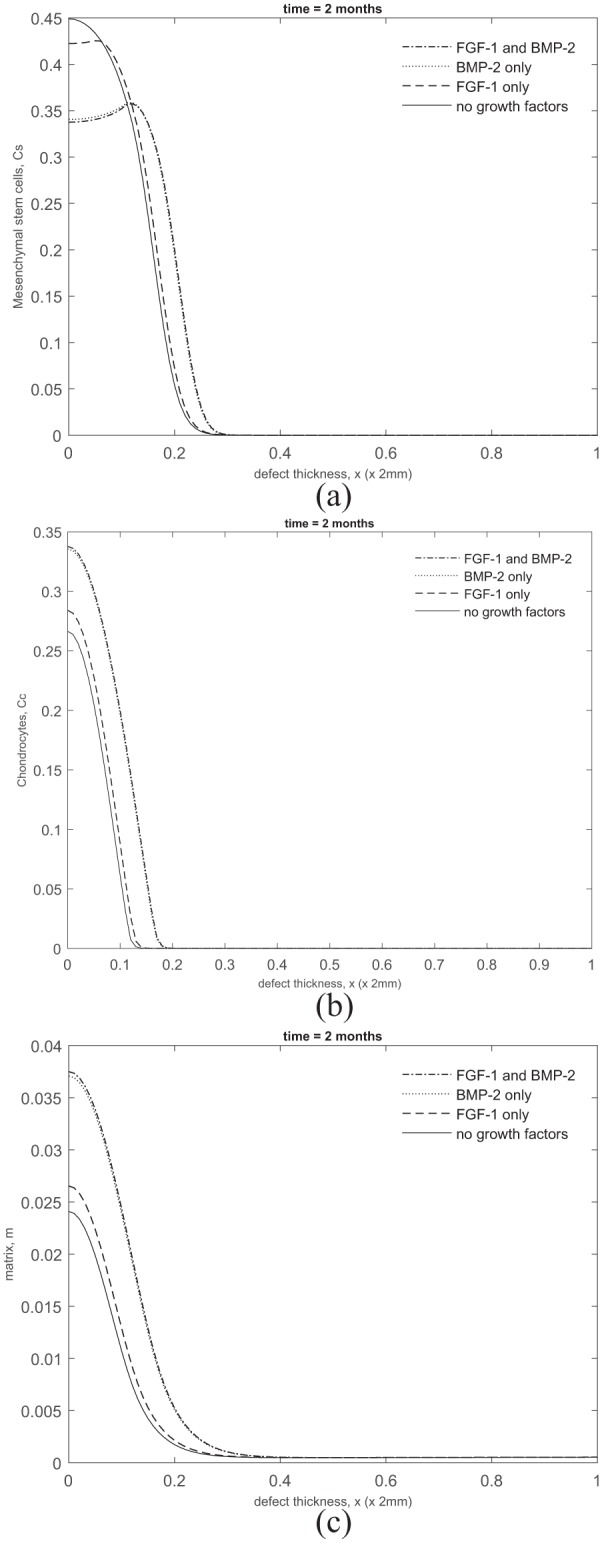
Comparison of (a) stem cell, (b) chondrocyte and (c) matrix densities at *t* = 2 months when including FGF-1 and BMP-2 (dot-dashed lines), BMP-2 alone (dotted lines), FGF-1 alone (dashed lines) and no growth factors (solid lines).

We now consider the influence of growth factors FGF-1 and BMP-2 on the evolution of the cell and matrix densities. We have an initial seeding of stem cells at the bottom of the defect with a small concentration of both the growth factors $\left({\bar{g}}_{init}={\bar{b}}_{init}=10^{-2}\right)$ and chondrocytes and matrix $\left({\bar{C}}_{C}^{\left(0\right)}={\bar{m}}_{3}=10^{-4}\right)$ pre-existing uniformly within the defect (Panel 1 in Figure 6). Similar to the early time behaviour observed in the previous case, the stem cells start to proliferate with a slow decline of nutrients in the first few days. These proliferating stem cells produce FGF-1 which gradually increases in concentration near the bottom of the defect. This has a minor influence on chondrocyte proliferation, though. The initial seeding of chondrocytes, however, produces sufficient BMP-2 which reduces the stem cell density threshold for differentiation into chondrocytes at an earlier time compared to the previous case. This resultant increase in the production of chondrocytes through stem cell differentiation in turn speeds up the matrix production process. This increase in chondrocyte and matrix density at early time (*t* = 2 months) is clearly observed in Panel 2 of Figure 6 (also see Figure 8(b) and (c) for comparison with the case when no growth factors are present). Also, at this time point, we already observe a diffusion front in the stem cell density starting to form (Panel 2 in Figure 6; also see Figure 8(a) for comparison with the case when no growth factors are present). The growth factor concentrations are much higher near the bottom of the defect owing to the higher density of stem cells and chondrocytes there (Panel 2 in Figure 6). The relative abundance of BMP-2 here, in particular, further lowers the threshold stem cell density to its minimum value, ${\bar{C}}_{S_{0\min}}$, which increases the chondrocyte density (compare the chondrocyte densities in Panel 2 in Figures 4 and 6). From 2 months onwards, we observe the two fronts in the stem cell and chondrocyte density to gradually migrate up the defect where a higher concentration of nutrients are available (Panel 3 in Figure 6 shows the evolution at *t* = 3 months). We note that these fronts are slightly ahead compared to those from the previous results (Panel 3 in Figure 4) at this time point. This is due to the diffusion fronts forming earlier for this case as described above. In addition, there is a larger volume of matrix in the defect at time points between 2 and 3 months (see Panels 2 and 3 in Figure 6; also see Figure 8(c) for comparison with the case when no growth factors are present). The evolution past 6 months shown in Panel 1 of Figure 7 is similar to the previous set of results, albeit with slightly higher levels of matrix at comparable time points. This might be due to the FGF-1 growth factor concentration enhancing the chondrocyte proliferation resulting in additional matrix. We note here that there is no contribution from stem cell differentiation since the stem cell density has fallen well below its minimum threshold density, ${\bar{C}}_{S_{0\min}}=0.315$, for differentiation into chondrocytes. For time 12 months and beyond, the matrix formation continues until the defect eventually fills up with matrix (see Panels 2 and 3 in Figure 7).

We now highlight the differences at early time observed in the cell and matrix densities in the two sets of simulations above. We pick a representative time point at *t* = 2 months to depict this. We will also look at cases where either FGF-1 or BMP-2 alone is included to determine which growth factor has a stronger influence, if at all, on the system. In Figure 8(a), we observe that the stem cell density near the bottom of the defect is lower when growth factors are included. This suggests that stem cell differentiation has occurred earlier for this case due to the lowering of the threshold density. The higher level of stem cell density for the case when growth factors are absent implies delay in stem cell differentiation due to the threshold density not being exceeded. Looking at the chondrocyte levels in Figure 8(b), we observe that including growth factors results in a slightly higher chondrocyte density near the bottom of the defect compared to that without growth factors. These additional chondrocytes are produced by stem cell differentiation which occurs earlier in the presence of growth factors. This increase in chondrocyte density results in a significantly larger amount of matrix being formed compared to that without growth factors (Figure 8(c)). Moreover, there is no discernible difference in the cell and matrix densities when comparing the cases where both growth factors are included to that where BMP-2 alone is included. This indicates that BMP-2 alone has a much more significant influence on the system than FGF-1 alone at least at early time. This is mainly due to the lowering of the threshold density for stem cell differentiation into chondrocytes. However, at later time, this influence gradually decreases as the stem cell density falls well below its minimum threshold density for differentiation. Past 4 months, the system then evolves similar to that without growth factors.

To further highlight the effects of FGF-1 and BMP-2 on the system, we show comparisons of the overall densities of chondrocytes, $C_{C}$; MSCs, $C_{S}$; and ECM, *m*, with and without the effects of growth factors over 24 months.

These results allow us to quantify the percentage difference between the cell and matrix types, with and without the effects of growth factors, enabling us to quantify our specific research question posed in the ‘Introduction’ section. This gives us an indication of how considering these growth factors in a co-culture will impact matrix deposition.

**Figure 9.**
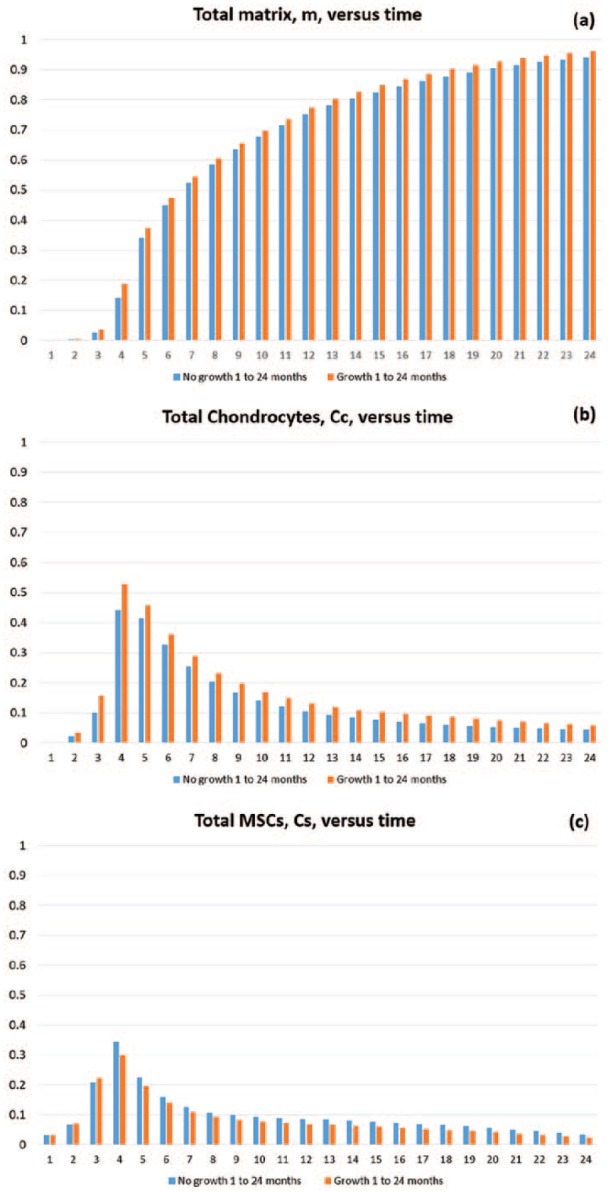
Mean densities of (a) matrix, *m*; (b) chondrocytes, *C_C_* ; and (c) MSCs, *C_S_*, as a function of the time, in months, from 1 to 24 months for simulations with (orange) and without (blue) growth factors.

Figure 9(a) shows overall matrix densities between 1 month and 2 years in time increments of 1 month. From this figure it is clear that the main difference in matrix densities is at early times, with effects seeming to subside after around 4–5 months. At 2 months, we have a 65% increase in matrix density when growth factors are included, declining to 34% increase at 4 months. From 4 months onwards, the percentage change of matrix density is still greater with growth factors, but decreases in magnitude.

Figure 9(b) shows the difference in chondrocyte levels within the defect up to 24 months. Chondrocyte proliferation and MSC differentiation into chondrocytes are mechanisms both effected by the growth factors, meaning we expect to see a pronounced increase in this cell type in the defect during healing. At 2 months, we see a 66% increase in chondrocyte levels, declining to only a 19% increase at 4 months. The main increase in overall chondrocyte densities is primarily observable up to 4 months and subsides thereafter.

Figure 9(c) shows MSC densities within the defect over 2 years. The stem cell differentiation into chondrocytes mechanism is directly effected by BMP-2, meaning we expect to see lower MSC levels in the defect at times that growth factors are most effective. At 1–3 months, we see a slight increase in overall MSC levels, but Figure 8(a) shows a diffusion front of MSCs forming sooner than without growth factors at 2 months due to the effects of FGF-1 and BMP-2. This indicates that MSC differentiation has been initiated sooner. In addition, at this time, chondrocyte densities are markedly higher than without growth factors (Figure 8(b)), meaning more BMP-2 is being produced. This implies evolution of MSCs is accelerated due to the effects of the growth factors during this time frame. At 4 months, we see a 13% decrease in MSC density, which is due to BMP-2 effects increasing due to increased chondrocyte densities around this time. After 4 months, a consistent trend of lower MSCs is observed in the defect for the case with growth factors.

These results indicate and validate that the time frame for FGF-1 and BMP-2 effects to be significant is at early times primarily up until 4 months. The effects of growth factors subside thereafter, as demonstrated by the similarity between Figures 5 and 7. The experiments of Wu.^13^ are in vitro and therefore performed over short periods of time. This therefore corroborates the effects they observe. It is likely that the effects of FGF-1 and BMP-2 decline due to other limiting factors in the model such as nutrient concentration and motility of cells (Figures 6 and 7).

### Sensitivity of parameters and initial conditions

The model is used to simulate a variety of parameter values and initial conditions. A sensitivity analysis will help in pinpointing those parameters that the system is sensitive to, which could indicate biological significance. In addition, a parameter whose value has been approximated and not deemed to be sensitive to change indicates that this approximate value is a good representation of that parameter value. Here, we only consider the sensitivity of the model to variations in the FGF-1 and BMP-2 parameters and initial conditions. These are described briefly in Table 3 and the ones which most influenced model results are discussed in detail below. The sensitivity to the other parameters and initial conditions are similar to that discussed by Lutianov et al.^17^ and we refer the reader to Table 3 in this article.

**Table 3.** Sensitivity of parameters.

Parameters	Sensitivity description
Initial FGF-1 concentration, ${\bar{g}}_{init}$	Increasing ${\bar{g}}_{init}$ results in a small increase in chondrocyte proliferation and matrix deposition at very early times; thereafter, no change is observable
Initial BMP-2 concentration, ${\bar{b}}_{init}$	Increasing ${\bar{b}}_{init}$ has no effect on the system since it degrades quickly before it has the chance to take effect; it starts being produced again when a sufficient level of chondrocyte density is reached to counteract its degradation
FGF-1 production constant, ${\bar{p}}_{9}$	Increasing ${\bar{p}}_{9}$ results in a minor increase in chondrocyte proliferation and matrix levels at early time; decreasing ${\bar{p}}_{9}$ decreases matrix levels marginally at early time; no noticeable difference thereafter
**BMP-2 production constant**, ${\bar{p}}_{12}$	See details in text
FGF-1 degradation rate, ${\bar{p}}_{11}$	Increasing/decreasing ${\bar{p}}_{11}$ has no significant change to cell density levels and evolution characteristics
**BMP-2 degradation rate**, ${\bar{p}}_{13}$	See details in text
FGF-1/BMP-2 diffusion coefficient, $D_{g,b}$	Increasing $D_{g,b}$ has no significant change to cell density levels and evolution characteristics
FGF-1/BMP-2 flux coefficient, $\bar{\gamma }$, $\bar{\chi }$	Increasing/decreasing $\bar{\gamma }$, $\bar{\chi }$ has no significant change to cell density levels and evolution characteristics
FGF-1 matrix deposition rate, ${\bar{p}}_{8_{00}}$	Increasing ${\bar{p}}_{8_{00}}$ up to 1 has minor effects to overall matrix levels We see higher matrix levels primarily at the bottom of the defect indicating main effect at early time. The general evolution remains unchanged and earlier healing time is not achieved
**Minimum threshold stem cell density**, ${\bar{C}}_{S_{0min}}$	See details in text
**Threshold stem cell density reduction factor**, $\bar{\alpha }$	See details in text
Stem cell differentiation rate, ${\bar{p}}_{2}$	Variations (including assumed dependency on BMP-2 concentration) only resulted in minor differences in cell and matrix densities and accelerated growth; general evolution characteristics remain unchanged

Those highlighted in bold are further described in the text.

We described earlier that the increased levels of chondrocyte and matrix densities observed at early time in the presence of growth factors were primarily due to the reduction in the threshold stem cell density for differentiation into chondrocytes (Figure 8(a)–(c)). We have further investigated variations in the parameters we found that this reduction was most sensitive to the BMP-2 growth factor production constant, ${\bar{p}}_{12}$; the BMP-2 degradation rate, ${\bar{p}}_{13}$; the minimum threshold stem cell density, ${\bar{C}}_{S_{0min}}$; and the threshold stem cell density reduction factor, $\bar{\alpha }$ (last function, equation (13)).

**Figure 10.**
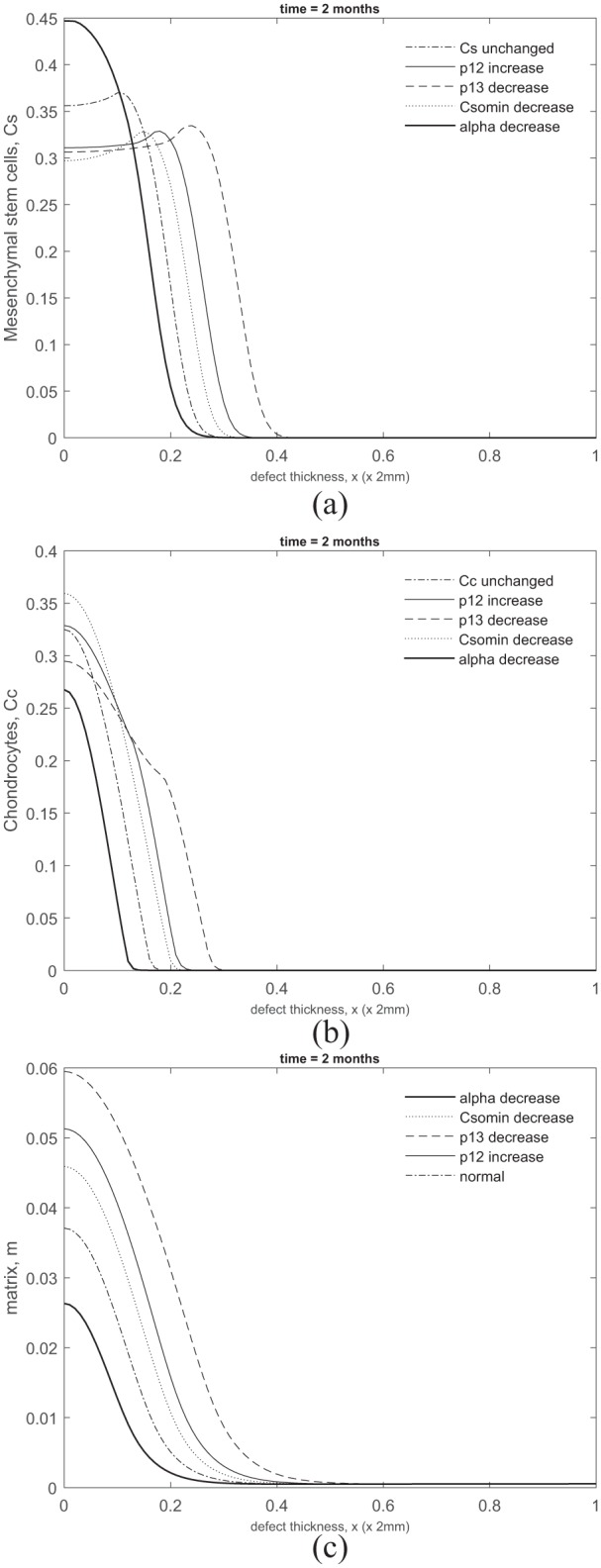
Comparison of (a) stem cell, (b) chondrocyte and (c) matrix densities at *t* = 2 months when varying the BMP-2 growth factor production constant, ${\bar{p}}_{12}$ (darker solid lines), the BMP-2 degradation rate, ${\bar{p}}_{13}$ (dashed lines), the minimum threshold stem cell density, ${\bar{C}}_{S_{0\min}}$ (dotted lines) and the threshold stem cell density reduction factor, $\bar{\alpha }$ (lighter solid lines) independently from their base values (dot-dashed lines). See text for parameter values used.

Figure 10(a)–(c) shows the stem cell, chondrocyte and matrix density, respectively, at *t* = 2 months by varying ${\bar{p}}_{12,13}$, ${\bar{C}}_{S_{0\min}}$ and $\bar{\alpha }$ independently from their base values. In the simulations shown, ${\bar{p}}_{12}=267$ (10-fold increase from its base value), ${\bar{p}}_{13}=0.154$ (100-fold decrease from its base value), ${\bar{C}}_{S_{0\min}}=0.28$ (reduces ${\bar{C}}_{S_{0}}$ by 20% in comparison with its base value which imposes a 10% reduction) and $\bar{\alpha }=1$ (100-fold decrease from its base value). Increasing ${\bar{p}}_{12}$ and decreasing ${\bar{p}}_{13}$ and ${\bar{C}}_{S_{0\min}}$ resulted in stem cell differentiation to occur much earlier in comparison with their base values (Figure 10(a)). Moreover, stem cell differentiation was most delayed when *α* was decreased. The chondrocyte density levels appeared less sensitive to variations in these parameters (Figure 10(b)). The diffusion of chondrocytes away from the defect was observed slightly earlier when ${\bar{p}}_{12}$ was increased, and ${\bar{p}}_{13}$ and ${\bar{C}}_{S_{0\min}}$ were decreased compared to the base values and when *α* was decreased. This was a consequence of the stem cell differentiation occurring earlier when these parameters were varied. The matrix density levels shown in Figure 10(c) show slightly enhanced levels compared to the base value and when *α* was decreased. This was again due to stem cell differentiation into chondrocytes occurring early and subsequently producing more matrix.

An alternative method to implement the effect of BMP-2 on stem cell differentiation is to vary the stem cell differentiation rate, ${\bar{p}}_{2}$ with the BMP-2 concentration, while keeping the threshold stem cell density, ${\bar{C}}_{S_{0}}$ fixed. As detailed in Table 3, we found no significant influence of this on the system and the model results appeared much less sensitive to variations in ${\bar{p}}_{2}$ than to the stem cell density threshold variation considered in this work.

Our sensitivity indicates that the values we have approximated are also not extremely sensitive to change and hence a good representation of that parameter value. Identifying the sensitive parameters from the sensitivity analysis could provide important information for in vitro studies, indicating which mechanisms need to be focused on or manipulated experimentally to produce a desired effect, such as increased cell and/or matrix densities.

## Discussion

We have extended the model proposed by Lutianov et al.^17^ to consider the influence of two growth factors, BMP-2 and FGF-1, on the regeneration of a cartilage defect. These two growth factors serve as examples that embody the type of interactions that can occur between MSCs and chondrocytes, which would typically affect stem cell and chondrocyte proliferation, differentiation and matrix production. The interactions in our model are those hypothesised by Wu,^13^ which they formulated on basis of their and others’ experimental data. Our simulations show that the interactions from the growth factors enhance matrix production at early times. This is consistent with in vitro results of Wu,^13^ whose findings show increased GAG levels in co-culture pellets of MSCs and chondrocytes up to 4 weeks after culture, indicative of increased matrix deposition. Of course, unlike the co-culture experiments which start with a mixture of stem cells and chondrocytes, our initial conditions represented implantation of only stem cells. However, once stem cells differentiated into chondrocytes in our model, they displayed the same stem cell–chondrocyte interaction observed in the co-culture experiments with similar trophic effects.^13^

Our model considered two cell types, MSCs and chondrocytes, and therefore studied the actions of the two growth factors within these restrictions. However, it is important to realise that the growth factors probably also play a role beyond these two cell types. Besides promoting chondrogenic differentiation of MSCs, BMP-2 can also induce chondrocyte hypertrophy and lead to endochondral ossification. FGF-1, along with other members of the FGF family, is thought to enhance collagen 1 expression leading to a fibrous cartilage being formed during chondral healing. It is hypothesised that when FGF-1 and BMP-2 are both present during the healing process, chondrocyte hypertrophy and fibrocartilage formation are not observed in the defect, indicating that FGF-1 suppresses the hypertrophy and BMP-2 inhibits the formation of fibrous cartilage.^13^ These functions indicate that both growth factors are involved in aspects of the healing process that we did not consider in this model, specifically chondrocyte hypertrophy and endochondral bone formation. In further work, we plan to extend our model to study these aspects. However, chondrocyte hypertrophy and endochondral bone formation have not been flagged as adverse effects after autologous stem cell or chondrocyte implantation to treat chondral defects, suggesting that with respect to the clinical application of these therapies, our model may be considered representative.^8^

Our model allowed us to investigate the influence of either growth factor, independent of the other. This enabled us to determine the sensitivity of stem cell–chondrocyte interaction to each growth factor. The results obtained when including only BMP-2 were very similar to those including both FGF-1 and BMP-2, both showing clearly increased matrix production at early time points. On the other hand, for the case where only FGF-1 was included, the matrix density levels at early times were increased only marginally when compared to the baseline case of no growth factors. This suggests that BMP-2 dominates the interaction and that the main positive effect of a mixture of the two cell types is due to enhanced chondrogenesis.

Our model found that the influence of chondrocytes on stem cell differentiation through BMP-2 affected the result more than the influence of stem cells on chondrocyte proliferation via FGF-1. This may be related to the effects of nutrient concentration in our model, which did influence chondrocyte proliferation but did not directly influence stem cell differentiation, though a knock-on effect would be expected from nurtient’s limiting effect on stem cell proliferation, but we would not expect this effect to be significant in our simulations due to a 100% MSC cell seeding. The lack of effect of FGF-1 could potentially indicate the initial growth factor concentration and rates we have selected from the literature are contentious, but our sensitivity analysis indicates that these parameters are not sensitive to change. This enables us to make the assumption our parameters are within a realistic biological range of which FGF-1 is effective. In our model, a lower nutrient concentration reduced or, if below the threshold nutrient concentration, completely stopped chondrocyte proliferation. We think this may explain why the effects of FGF-1 were relatively small, because in all our simulations, the nutrient concentration seems to be the main limiting factor. That nutrient concentration influences chondrocyte proliferation and has been demonstrated experimentally.^25^ In contrast, stem cell differentiation was not affected by nutrient concentration in our model, and as a result, a low nutrient concentration did not inhibit the effects of BMP-2. We are not aware of experimental studies addressing effects of nutrition on MSC *differentiation* into chondrocytes, but one study extensively addressed this issue related to osteoblast differentiation.^26^ This study concluded that during three-dimensional (3D) micromass culture (a situation comparable to the one in our model) osteoblast differentiation was not affected by nutrition but was a function of cell–cell contacts and cell–cell communication, exactly the phenomena we included in our model.

In our model, the influence of BMP-2 on stem cell differentiation was implemented through a lowering of the threshold stem cell density $C_{S_{0}}$ as a function of BMP-2 concentration. An alternative implementation would be through the stem cell differentiation rate, in a manner similar to our implementation of the influence of FGF-1 on chondrocyte proliferation. We compared both approaches in a sensitivity study and found no clear differences between them. In our sensitivity analysis, we found $p_{12},p_{13},C_{S_{0\min}},\;\mathrm{and}\;\alpha$ to be the most sensitive parameters in our model, which is discussed in detail in the ‘Sensitivity of parameters and initial conditions’ section. Despite a handful of our variables being approximated, our sensitivity indicates that these parameters are not significantly sensitive to change, indicating our values are a good representation of the parameter values we needed for the model but could not find data for.

Our model used two specific growth factors, BMP-2 and FGF-1, to investigate the interactions between MSCs and chondrocytes during cartilage repair following cell implantation. However, we should stress that our results are not limited to these two. We see these two growth factors as examples of how such interactions could occur. For instance, some experiments have found evidence that the influence from chondrocytes on stem cell differentiation acts via direct cell–cell contact instead of soluble factors or that other growth factors might be involved (see the work by Wu^13^ for an overview). A similar situation exists in relation to the influence of stem cells on chondrocyte proliferation. Nevertheless, whatever the precise mode through which the interaction occurs, the main aspect will always be that chondrocytes influence stem cell differentiation and stem cells influence chondrocyte proliferation. Although our model may therefore not capture all details, it does certainly capture the gist of the interaction between the two cell types and we therefore think that its broad conclusions are still relevant if the details may be incorrect.

Our model enables us to better understand the underlying mechanisms taking place during chondral healing when we consider the effects of growth factors. This model can be used as an informative tool for clinicians and experimentalists alike, giving insight into the effects of the growth factors FGF-1 and BMP-2 on chondrocyte proliferation and MSC differentiation. This work provides insight regarding the clinical significance of the mechanisms involved in the FGF-1–BMP-2 feedback loop without requiring experimentation, also enabling us to identify with ease the most effective growth factor in our model. Our sensitivity analysis demonstrates that increasing FGF-1 and BMP-2 will have minor effect due to limiting factors such as nutrient concentration and growth factor degradation. Our results also provide corroboration for experimental work already undertaken.^13^

The consideration of growth factors and their mediating influence on cell-to-cell interactions is an important step towards looking at more complex models such as implantations of a mixture of cells. The work by Wu^13^ shows how inserting a mixture of stem cells and chondrocytes together into a defect can promote matrix deposition, and therefore a faster healing time due to the trophic effects growth factors such as BMP-2 and FGF-1 have on the system. This currently is being investigated and will be published in the second part of this article.
